# Long-Term Effects of Organo-Mineral Fertilization on Floristic Composition and Biodiversity in High Nature Value Mountain Grasslands of the Apuseni Mountains (Romania)

**DOI:** 10.3390/plants15020271

**Published:** 2026-01-16

**Authors:** Ioana Ghețe, Claudiu Șerban, Alexandru Ghețe

**Affiliations:** 1 Department of Grasslands and Forage Crops, Faculty of Agriculture, University of Agricultural Sciences and Veterinary Medicine Cluj-Napoca, Calea Mănăstur 3-5, 400372 Cluj-Napoca, Romania; 2 Research and Development Institute for Montanology, 557085 Cristian, Romania; 3 Department of Technical and Soil Sciences, Faculty of Agriculture, University of Agricultural Sciences and Veterinary Medicine Cluj-Napoca, Calea Mănăstur 3-5, 400372 Cluj-Napoca, Romania; alexandru.ghete@usamvcluj.ro

**Keywords:** fertilizer gradient, organo-mineral input, High Nature Value (HNV) grasslands, biodiversity, long-term experiment, mountain grasslands, sustainable grassland management

## Abstract

This study evaluated the long-term effects of organo-mineral fertilization on floristic diversity, species diversity, and vegetation structure in an HNV grasslands of the Apuseni Mountains. The experiment included five fertilization variants (control, organic, organo-mineral, mineral, and intensive organo-mineral), applied over a period of more than 15 years. Floristic diversity was assessed using a modified Braun–Blanquet method and multivariate methods—cluster analysis, principal coordinate analysis (PCoA), MRPP procedure, and indicator species analysis (ISA). Our analysis showed a trophic gradient, from oligotrophic *Festuca rubra* grasslands to mesotrophic (*Agrostis capillaris–Trisetum flavescens*) and eutrophic (*Agrostis capillaris–Centaurea pseudophrygia*) communities, depending on the intensity of organo-mineral fertilization applied. Moderate organo-mineral fertilization maintained a balanced floristic diversity and higher Shannon and Simpson indices compared to variants fertilized only with mineral inputs. Organo-mineral inputs improved soil fertility and ecosystem resilience, supporting soil microbiota activity and reducing nutrient losses. Intensive mineral fertilization led to a reduction in floristic richness and the dominance of nitrophilic species. This study demonstrates that moderate organo-mineral fertilization (≤10 t ha^−1^ manure combined with N_50_P_25_K_25_) provides an optimal balance between grassland productivity and biodiversity conservation, offering practical guidance for the sustainable management of High Nature Value mountain grasslands.

## 1. Introduction

High Nature Value (HNV) grasslands are key components of the European rural landscape, recognized for their high biodiversity, ecological functions, and cultural and economic importance [1,2,3]. These semi-natural grassland systems support a wide range of ecosystem services, including soil conservation, carbon sequestration, hydrological regulation, and the preservation of traditional cultural landscapes [4,5,6]. At the same time, the European Union supports High Nature Value (HNV) grasslands, which occupy significant areas, particularly in mountainous and hilly regions, where extensive farming practices and low-input fertilization—typically involving nutrient additions below 50 kg N ha^−1^ year^−1^—have enabled the long-term (decadal-scale) conservation of floristic diversity [7,8,9]. This conservation effect is primarily linked to reduced nutrient availability, which limits competitive exclusion by fast-growing grasses and allows the persistence of stress-tolerant and oligotrophic species characteristic of semi-natural mountain grasslands. In Romania, grassland ecosystems are particularly widespread in the Carpathian Mountains, where they constitute nuclei of high natural value agriculture and are part of Natura 2000, playing an important role in biodiversity conservation and in maintaining mountain agro-cultural identity [10,11,12,13]. In the Apuseni Mountains, previous research has shown that maintaining the biodiversity of oligotrophic grasslands depends on traditional agricultural practices and the use of reduced organic inputs [14,15,16]. In parallel, the continuation of traditional management practices on the grasslands of the Apuseni Mountains has shaped distinctive landscape elements that define the identity of the cultural landscape [17,18,19]. Grassland ecosystems provide multiple ecosystem services that support both ecological functioning and the rural mountain economy. As provisioning ecosystem services, these grasslands supply high-quality cattle fodder with high protein and mineral content [20,21,22], but also valuable medicinal plants, such as *Arnica montana*, an emblematic species of the Apuseni Mountains [23,24,25,26] along with other plant resources of local interest, traditionally used in mountain households [27,28,29].

Through carbon sequestration and water regime regulation, these grassland ecosystems deliver ecosystem services that contribute to climate change mitigation [30,31,32,33]. At the same time, traditionally managed mountain grasslands are important for preventing soil erosion and stabilizing slopes, contributing to the adaptation of mountain ecosystems to climate change [34,35,36]. At the same time, these grassland ecosystems ensure the maintenance of soil fertility and nutrient cycling, promoting the resilience of agroecosystems through a balance between production and conservation [37,38]. HNV grasslands have distinct cultural and social value, being appreciated for their characteristic landscapes, for the conservation of agro-pastoral traditions, and for the high tourism potential of mountain areas [39,40,41]. In this context, these grassland ecosystems face a range of anthropogenic and environmental pressures that may compromise ecological stability and ecosystem functions. Agricultural intensification, through the excessive use of mineral fertilizers and mechanization, leads to the simplification of the vegetation cover, increased dominance of competitive grasses, and reduced biodiversity [42,43,44,45].

Conversely, agricultural abandonment triggers secondary successional processes, leading to woody species establishment and reduced forage quality [46,47,48,49,50]. This transition leads to a decrease in species richness and profound changes in the structure of plant communities, with the loss of economically valuable herbaceous plant species in favor of plants with high ecological plasticity, particularly fast-growing mesophilic and nitrophilous species (e.g., *Agrostis capillaris*, *Trisetum flavescens*, *Taraxacum officinale*), characterized by high nutrient uptake capacity, rapid biomass accumulation, and broad tolerance to increased soil fertility [51,52,53,54]. On a continental scale, the abandonment of grasslands without management interventions favors the invasion of woody species and the closure of landscapes [55,56], leading to significant habitat loss and associated biodiversity degradation [57,58]. At the same time, climate change acts as a multiplier, altering water and temperature regimes and accelerating ecological transition processes [59,60]. In addition, socio-economic transformations and land use changes profoundly influence the adaptive capacity of cultural landscapes in the Carpathian Mountains [60,61] and other European mountain regions, reducing the connectivity of traditional agro-pastoral systems [62,63,64]. Organo-mineral fertilization offers a sustainable alternative to exclusively mineral fertilization [38], allowing a balance to be maintained between productivity and the conservation of grassland ecosystem biodiversity [65,66], reducing greenhouse gas emissions, stabilizing the soil, without compromising the forage harvest [67,68]. A comparison of research focusing on fertilization systems in Romanian grasslands recommends mixed models for better sustainability of grassland ecosystems [69,70]. A global analysis shows the increased adoption of organo-mineral fertilizers in response to the pressure to combine agricultural efficiency with environmental protection [71,72]. Recent studies have shown that the combined application of organic and mineral fertilizers leads to significant increases in production and improved feed quality, even in mountain grasslands dominated by *Nardus stricta* [44,73,74]. Overall, organo-mineral fertilization allows for the conservation of biodiversity and the optimization of grassland ecosystem services [75], providing a balance between forage production needs and environmental requirements [76,77]. Although mineral fertilizers contribute to increased productivity and improved nutritional value of fodder [78,79], their application in high or repeated doses leads to a decrease in floristic diversity [37], favoring nitrophilic species and reducing the long-term stability of grassland ecosystems [80,81]. In contrast, the application of organo-mineral inputs supports biological processes in the soil, favoring balanced biomass growth and maintaining species characteristic of HNV grasslands [82,83]. The effects of organo-mineral fertilization are not limited to biomass production but also involve changes in soil chemical properties, nutrient availability, and microbial-mediated processes. Such effects can influence functional processes such as nutrient cycling, soil organic matter stabilization, and plant–soil interactions [84,85].

Long-term experiments are essential tools for identifying the critical ecological thresholds at which grassland ecosystems change their structure and functionality [86,87,88]. In the case of HNV grasslands in mountain areas, these studies are indispensable for assessing the impact of fertilization practices—especially organo-mineral inputs—on biodiversity, productivity, and ecosystem stability. The results obtained in such long-term experiments provide valuable and reliable scientific benchmarks for defining optimal fertilization levels that are compatible with the principles of sustainable agriculture [89,90,91]. At the same time, the integration of quantitative vegetation analysis methods, together with management approaches directly related to this study [92,93,94] such as organo-mineral fertilization strategies, mulching, and fractional application of inputs [95,96], contributes to a more robust assessment of grassland ecosystem functioning in mountain environments. These approaches allow the correlation of floristic and edaphic processes with the dynamics of climatic factors, providing an integrated view of the resilience of organo-mineral fertilized grasslands. Within the European policy framework, the management of HNV grasslands is strongly guided by environmental regulations aimed at limiting nutrient inputs and conserving biodiversity. In particular, the Nitrates Directive sets upper thresholds for nitrogen application and promotes practices that reduce nutrient leaching, while the Natura 2000 Habitats framework requires the maintenance of favorable conservation status for semi-natural grassland habitats. Consequently, fertilization in mountain HNV grasslands is restricted to low-intensity inputs and carefully controlled application regimes, which directly frame the type and intensity of organo-mineral fertilization treatments assessed in the present study.

The objective of this study is to assess the long-term effects of organo-mineral fertilization on the structure, diversity, and functioning of High Nature Value grasslands in the Apuseni Mountains, based on vegetation data collected after 15 years of continuous treatment application at the plot scale (20 m^2^). Specifically, the study aims: (i) to quantify changes in floristic composition and plant diversity along a gradient of fertilization intensity, using species richness and standard α-diversity indices (Shannon, Simpson, and Evenness); (ii) to compare vegetation structural responses under organic, mineral, and organo-mineral fertilization regimes, with vegetation structure defined in terms of species dominance patterns, relative cover, and community composition derived from floristic surveys and multivariate analyses; and (iii) to identify fertilization thresholds that balance forage productivity and biodiversity conservation, where thresholds are interpreted as ecological limits beyond which marked shifts in plant community composition and diversity occur. In this context, the balance between productivity and conservation is defined by the maintenance of species-rich grassland communities characteristic of HNV systems while avoiding floristic simplification associated with eutrophication. The results obtained can serve as scientific support for optimizing agro-ecological practices in sensitive areas of the Apuseni Mountains and for adopting them in line with the requirements of the Nitrates Directive and Natura 2000 framework. Despite extensive research on fertilization effects in grassland ecosystems, the long-term ecological responses of HNV grasslands to organo-mineral fertilization remain insufficiently documented, particularly with respect to thresholds that balance productivity and biodiversity conservation. In this study, we address this gap by testing whether moderate organo-mineral inputs can maintain floristic diversity and community stability while avoiding the trophic shifts associated with intensive fertilization. We hypothesize that fertilization intensity generates distinct and consistent ecological responses in HNV mountain grasslands, with identifiable ecological thresholds along the nutrient gradient. Specifically, we expect that low to moderate organo-mineral inputs (≤10 t ha^−1^ manure combined with up to 50 kg N ha^−1^ year^−1^) maintain species-rich grassland communities, whereas fertilization levels exceeding 100 kg N ha^−1^ year^−1^ promote marked shifts toward mesotrophic and eutrophic vegetation types characterized by reduced floristic diversity. In this context, “predictable” responses refer to consistent changes in floristic composition and diversity patterns observed across treatments, rather than to formal statistical or mechanistic modeling. The optimal management level is defined ecologically as the fertilization regime that preserves high α-diversity and community structure characteristic of High Nature Value grasslands, while avoiding floristic simplification associated with intensive nutrient inputs. In this study, fertilization thresholds are quantitatively defined by nutrient input levels of ≤50 kg N ha^−1^ year^−1^, associated with the maintenance of high species richness and diversity indices, and ≥100 kg N ha^−1^ year^−1^, beyond which pronounced shifts toward mesotrophic and eutrophic plant communities and significant reductions in α-diversity were observed. These thresholds are validated by consistent changes in species richness, Shannon and Simpson indices, as well as by clear separation of treatments in multivariate analyses (PCoA, MRPP, and Indicator Species Analysis).

## 2. Results

The long-term fertilization experiment was established in 2001, the vegetation data analyzed in this study were collected in 2015. These data capture the cumulative effects of 15 consecutive years of fertilization on grassland floristic composition and structure, reflecting stabilized community responses to sustained nutrient inputs under consistent management conditions.

### 2.1. Cluster Analysis of Vegetation Under the Influence of Organo-Mineral Fertlization

**Figure 1:**
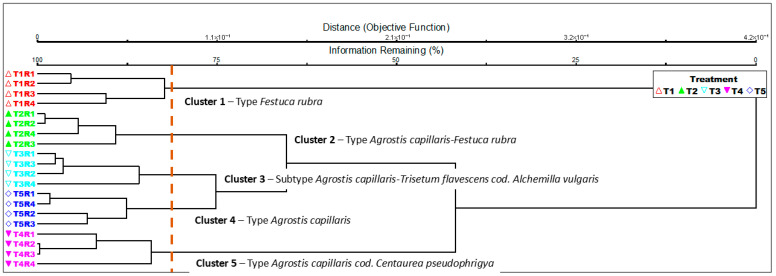
Dendrogram of plant community types. Legend. T1—control variant—*Zero-input*; T2—10 t ha^−1^ manure—*Low-input organic*, T3—10 t ha^−1^ manure + N_50_P_25_K_25_—*Medium-input organic mineral*, T4—N_100_P_50_K_50_—*Medium-input mineral*, T 5—10 t ha^−1^ manure + N_100_P_50_K_50_—*High-input*; R1, R2, R3, R4 = the four replications.

The application of organo-mineral fertilization caused major changes in the floristic composition of the grassland. Cluster analysis (Sørensen-Bray–Curtis index, UPGMA method) allowed for the clear separation of experimental areas into five distinct vegetation groups, corresponding to an ecological gradient determined by fertilization intensity (Figure 1). Cluster 1 (*Festuca rubra* grassland type) includes all control variants (T1R1–T1R4), characterized by the dominance of the *Festuca rubra* species. This group represents unfertilized mountain grassland, typically oligotrophic, stable phytocenosis, with high floristic diversity and a large proportion of perennial, stress-tolerant species. Cluster 2 (*Agrostis capillaris*–*Festuca rubra* grassland type) represents all variants with low litter input (T2R1–T2R4). Moderate organic fertilization (10 t ha^−1^) stimulated the emergence of mesotrophic species, but *Festuca rubra* remained codominant. This grouping of plant communities shows a transition phase towards communities with slightly increased productivity, but still ecologically balanced. Cluster 3 (Subtype *Agrostis capillaris*–*Trisetum flavescens* cod. *Alchemilla vulgaris*) corresponds to the variant with medium input of organic-mineral inputs (T3R1–T3R4). The presence of *Trisetum flavescens* and *Alchemilla vulgaris* confirms that the phytocenosis is mesotrophic, with a balanced structure between diversity and productivity. Cluster 4 (*Agrostis capillaris* grassland type) includes variants fertilized only with mineral inputs (T4R1–T4R4), in which the species *Agrostis capillaris* becomes dominant. This type of plant community shows a tendency toward a decrease in floristic composition and an increase in the proportion of nitrophilous plant species. Cluster 5 (*Agrostis capillaris* cod. *Centaurea pseudophrygia*) is represented by all variants with high fertilization inputs (T5R1–T5R4), where there are communities of plants with highly productive mesophilic species and the species *Centaurea pseudophrygia* appears codominant, with a more uniform floristic composition and reduced floristic diversity [37,97].

The results of the cluster analysis were also confirmed by the PCoA analysis, which highlights the same floristic groupings corresponding to the trophic gradient determined by the organo-mineral fertilization inputs.

### 2.2. Spatial Distance in Plant Community Projection Due to Long-Term Fertilization Organo-Mineral

**Figure 2:**
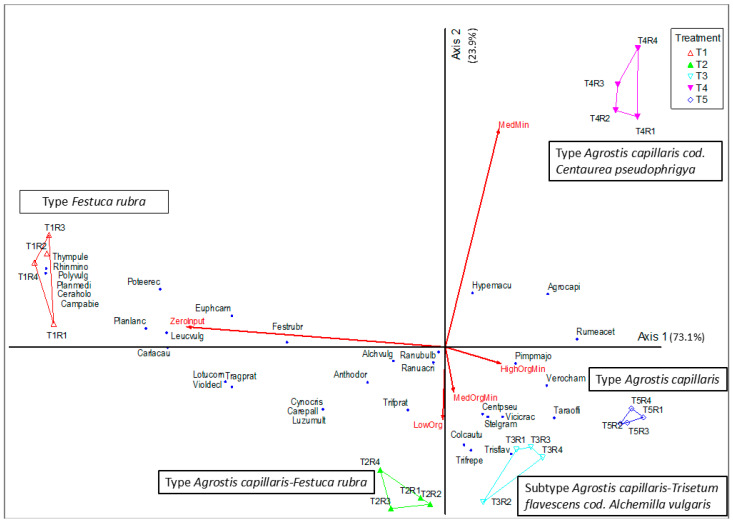
PCoA of grassland types modified by the application of orgao-mineral inputs (*Principal Coordinates Analysis (PCoA*) based on *Bray–Curtis* distance showing the separation of treatments according to fertilization intensity. Ordination axes are scaled and labeled with the percentage of variance explained (PCoA1 = 73.1%, PCoA2 = 23.9%). Control (T1) and low-input organic (T2) plots grouped on the negative side of Axis 1, while mineral and organo-mineral variants (T3–T5) clustered on the positive side, reflecting a trophic gradient from oligotrophic to eutrophic grassland types) *Legend*: *Type*—Type of grassland; *Subtip*—grassland subtype; T1—control variant (unfertilized), T2 —10 t/ha^−1^ manure, T3—10 t/ha^−1^ manure + N_50_P_25_K_25_, T4—N_100_P_50_K_50_, T5—10 t/ha^−1^ manure + N_100_P_50_K_50_; R1, R2, R3, R4 = the four replications; *ZeroInput*—T1-control variant—*Zero-input*; *LowOrg*—T2—10 t/ha^−1^ manure—*Low-input* organic, *MedOrgMin*—T3—10 t/ha^−1^ manure + N_50_P_25_K_25_—*Medium-input* organo-mineral, *MedMin*—T4—N_100_P_50_K_50_—*Medium-input* mineral, *HighOrgMin*—T5—10 t/ha^−1^ manure + N_100_P_50_K_50_—*High-input. Achillea millefolium* (Achimill), *Agrostis capillaris* (Agrocapi), *Alchemilla vulgaris* (Alchvulg), *Anthoxanthum odoratum* (Anthodor), *Briza media* (Brizmedi), *Campanula patula* (Camppatu), *Carex pallescens* (Carepall), *Carlina acaulis* (Carlacau), *Centaurea pseudophrygia* (Centpseu), *Cerastium glom-eratum* (Ceraglom), *Colchicum autumnale* (Colcautu), *Crepis biennis* (Crepbien), *Festuca pratensis* (Festprat), *Festuca rubra* (Festrubr), *Gentiana lutescens* (Gentlute), *Gymnadenia conopsea* (Gymncono), *Hieracium aurantiacum* (Hieraura), *Hypericum maculatum* (Hypemacu), *Leontodon autumnalis* (Leonautu), *Leucanthemum vulgare* (Leucvulg), *Lotus corniculatus* (Lotucorn), *Luzula multiflora* (Luzumult), *Pimpinella major* (Pimpmajo), *Plantago lanceolata* (Planlanc), *Plantago media* (Planmedi), *Polygala vulgaris* (Polyvulg), *Potentilla erecta* (Poteerec), *Ranunculus bulbosus* (Ranubulb), *Ranunculus acris* (Ranuacri), *Rhinanthus minor* (Rhinmino), *Rumex acetosella* (Rumeacet), *Scabiosa columbaria* (Scabcolu), *Stellaria graminea* (Stelgram), *Taraxacum officinale* (Taraoffi), *Thymus pulegioides* (Thympule), *Tragopogon pratensis* (Tragprat), *Trifolium repens* (Trifrepe), *Trifolium pratense* (Trifprat), *Trisetum flavescens* (Trisflav), *Veronica chamaedrys* (Verocham), *Vicia cracca* (Vicicrac) and *Viola declinata* (Violdecl).

The PCoA (Principal Coordinates Analysis) analysis, performed based on the Bray–Curtis distance, showed a clear separation of the experimental variants according to the intensity of fertilization (Figure 2). The first two ordinal axes together explain 97% of the total variation in floristic composition, confirming the relevance of the trophic gradient in structuring plant communities (Table 1), axis 1 being the most important.

The main ecological gradient, corresponding to the level of mineral inputs, was outlined on axis 1 (73.1%). The control variants (*ZeroInput*) and those with reduced organic fertilization (*LowOrg*) are grouped on the negative side of the axis, being associated with species characteristic of oligotrophic grasslands, such as *Festuca rubra*, *Carex pallescens*, *Luzula multiflora* and *Trifolium pratense*. In contrast, the medium and high fertilization variants (*MedMin* and *HighOrgMin*) are on the positive side, correlated with the species *Agrostis capillaris*, *Trisetum flavescens*, *Hypericum maculatum* and *Centaurea pseudophrygia*, indicating a mesotrophic to eutrophic plant community.

Axis 2 (23.9%) describes secondary variations associated with differences between organic and organo-mineral fertilization types. The *MedOrgMin* and *LowOrg* variants occupy an intermediate position, confirming a transitional floral structure between preserved communities and those with predominantly mineral fertilization. Beyond the main trophic gradient expressed by Axis 1, Axis 2 reflects differences in plant community composition related to the fertilization regime. This axis distinguishes treatments managed with organic or organo-mineral inputs from those receiving exclusively mineral fertilization, indicating that vegetation responses are influenced not only by nutrient intensity but also by the form of fertilization. Consequently, Axis 2 captures a management-driven fertilization gradient that complements the primary intensity gradient.

The association of experimental vectors with ordinal axes (Table 2) shows a significant negative correlation between axis 1 and the *ZeroInput* (T1) treatment (r = −0.922, *p* < 0.001), respectively, positive correlations between the same axis and *MedMin* treatments (r = 0.419, *p* < 0.05) and *HighOrgMin* (r = 0.427, *p* < 0.05). These relationships confirm that the main gradient of variation (Axis 1) reflects the intensification of fertilization and the floristic transition from oligotrophic to mesotrophic and eutrophic grasslands. The axis shows secondary variations mainly related to the type of fertilization. The association is strongly positive for the mineral treatment *MedMin*/T4 (r = 0.846, *p* < 0.001), while *LowOrg*/T2 (r = −0.487, *p* < 0.01) and *MedOrgMin*/T3 (r = −0.385, *p* < 0.05) show negative associations, indicating the difference between mineral and organic/organo-mineral input on this axis. *HighOrgMin*/T5 has a slightly negative association (r = −0.233; insignificant), and the *ZeroInput*/T1 control has a modest positive association (r = 0.258; insignificant). PCoA analysis, T4 treatments are on the positive side of Axis 2, T2–T3 on the negative side, and T1 positions close to the center. This model shows that Axis 2 separates the type of fertilization (mineral vs. organic/organo-mineral), while Axis 1 orders the intensity of the trophic gradient.

The results are consistent with the cluster analysis, both methods indicating a succession from oligotrophic *Festuca rubra* type of grasslands to plant communities such as mesotrophic *Agrostis capillaris–Trisetum flavescens* and eutrophic *Agrostis capillaris–Centaurea pseudophrygia*. This ranking highlights the ecological gradient from preserved grassland systems to intensified systems, generated by the increasing input of nutrients.

MRPP analysis (Table 3) confirmed the existence of significant differences in floristic composition between all experimental treatment variants. Negative values in statistics T (between −4.35 and −4.47) indicates a clear separation between the experimental groups, and the positive values of coefficient A (0.42–0.74) reflect a high degree of homogeneity within each group of plant communities. All comparisons between treatments were statistically significant (*p* < 0.01), showing that each fertilization level—organic, organo-mineral, or mineral—generated a distinct floristic composition with consistent ecological differences. The strongest separation was recorded between the control variant (T1) and the mineral-fertilized variants (T4; A = 0.741) or organo-mineral variants (T3; A = 0.703), indicating that nitrogen and phosphorus fertilization causes the greatest changes in the structure of plant communities. Also, the differences between variants T2 (reduced organic fertilization) and T3–T4 were significant, but with lower values of coefficient A, indicating a gradual transition between oligotrophic and mesotrophic phytocenoses.

The separation of the fertilized variants with high-intensity inputs (T4 and T5) had a lower A coefficient (0.593), indicating a tendency toward floristic homogenization at high input levels. The MRPP analysis validates the results obtained through cluster and PCoA, confirming that the intensification of fertilization drives an ordered ecological succession, from oligotrophic *Festuca rubra* grasslands toward plant communities such as the mesotrophic *Agrostis capillaris–Trisetum flavescens* and the eutrophic *Agrostis capillaris–Centaurea pseudophrygia*.

### 2.3. The Response of Plant Species to Organo-Mineral Inputs

The correlation analysis between the dominant plant species and the ordination axes (Table 4) highlights a clear trophic gradient associated with Axis 1, which separates species characteristic of oligotrophic grasslands from those of mesotrophic and eutrophic conditions. On Axis 1, positive values of the r coefficient correspond to mesotrophic and nitrophilic species, characteristic of mineral and organo-mineral fertilization treatments, such as *Agrostis capillaris* (r = 0.787, *p* < 0.001), *Trisetum flavescens* (r = 0.300, *p* < 0.05), *Rumex acetosa* (r = 0.653, *p* < 0.01), *Taraxacum officinale* (r = 0.617, *p* < 0.01), *Hypericum maculatum* (r = 0.291, *p* < 0.05) and *Veronica chamaedrys* (r = 0.728, *p* < 0.01). These species define mesotrophic–eutrophic communities, developed on soils with high nitrogen and phosphorus availability, resulting mainly from combined organic-mineral fertilization. Species with strongly negative *r*-values are associated with oligotrophic, unfertilized or only slightly fertilized grasslands, such as *Festuca rubra* (r = −0.952, *p* < 0.001), *Carex pallescens* (r = −0.691, *p* < 0.001), *Luzula multiflora* (r = −0.691, *p* < 0.001), *Lotus corniculatus* (r = –0.828, *p* < 0.001), *Alchemilla vulgaris* (r = −0.507, *p* < 0.01), *Carlina acaulis* (r = −0.840, *p* < 0.001) and *Thymus pulegioides* (r = −0.922, *p* < 0.001). These species are sensitive to increases in soil fertility, indicating the maintenance of the HNV grassland character in the control and low-input organic variants.

On Axis 2, secondary variation patterns related to the type of fertilization can be observed. Significant positive correlations were recorded for *Hypericum maculatum* (r = 0.826, *p* < 0.001) and *Agrostis capillaris* (r = 0.584, *p* < 0.01), indicating the preference of these species for mineral and organo-mineral fertilization treatments. Negative correlations characterize species associated with organic fertilization, such as *Trifolium repens* (r = −0.783, *p* < 0.001), *Trifolium pratense* (r = −0.503, *p* < 0.01), *Centaurea pseudophrygia* (r = −0.742, *p* < 0.001), *Colchicum autumnale* (r = −0.887, *p* < 0.001) and *Stellaria graminea* (r = −0.576, *p* < 0.01). This distribution confirms that Axis 1 reflects the intensity of fertilization, whereas Axis 2 differentiates the type of inputs applied (organic vs. mineral). From an ecological perspective, the results indicate a floristic transition from oligotrophic *Festuca rubra*-type grasslands toward mesotrophic *Agrostis capillaris–Trisetum flavescens* plant communities, dominated by species adapted to more fertile and productive soils.

The additional analysis of correlations among plant communities revealed patterns consistent with those obtained through ISA and PCoA, confirming the positive associations of mesotrophic species under moderate fertilization treatments and the clear separation of oligotrophic species in the control variants. This supports the ecological coherence of the identified trophic gradient.

### 2.4. Indicator Species Analisys to the Gradient of Applied Inputs Organo-Mineral

The ISA analysis (Table 5) highlighted clear groups of indicator species corresponding to each fertilization level, confirming the trophic gradient previously identified through ordination and cluster analysis. For the control variant (T1—*Zero-input*), the highest number of species with significant indicator values (*p* < 0.01) was identified, including *Festuca rubra*, *Campanula abietina*, *Cerastium holosteoides*, *Plantago media*, *Potentilla erecta*, *Thymus pulegioides* and *Rhinanthus minor*. These species are characteristic of oligotrophic, low-fertility mountain grasslands, preserving the traditional floristic composition and the biodiversity typical of HNV grasslands.

In the variants with low-level organic fertilization (T2—*Low-input*), species such as *Trifolium pratense* and *Trifolium repens* (*p* < 0.05) were associated, indicating a moderate increase in soil fertility and enhanced productivity without a complete loss of floristic diversity. These legume species contribute to the natural enrichment of nitrogen, supporting the ecological balance of the grassland ecosystem. For the medium organo-mineral fertilization variant (T3—*MedOrgMin*), species such as *Trisetum flavescens* and *Alchemilla vulgaris* (*p* < 0.05) were highlighted, mesotrophic species that prefer soils with moderate fertility. They define transitional plant communities, typical of productive grasslands, but still ecologically stable. In the mineral fertilization variant (T4—*MedMin*), indicator species such as *Agrostis capillaris* and *Hypericum maculatum* (*p* < 0.01) were identified, characterizing mesotrophic–eutrophic plant communities dominated by highly productive perennial grasses. For the high organo-mineral input treatment (T5—*HighOrgMin*), species such as *Taraxacum officinale*, *Veronica chamaedrys*, *Pimpinella major* and *Centaurea pseudophrygia* were identified, which are typical of eutrophic grasslands with reduced floristic diversity and a predominance of nitrophilous elements.

The ISA analysis confirms a directed ecological succession: from oligotrophic *Festuca rubra* plant communities (unfertilized) to mesotrophic *Agrostis capillaris–Trisetum flavescens* types and eutrophic *Agrostis capillaris–Centaurea pseudophrygia* communities as fertilization intensity increases. These results are in strong agreement with those obtained through PCoA and MRPP, emphasizing the coherence of the fertilization-induced trophic gradient in the structure of HNV mountain vegetation.

### 2.5. Effects of Organo-Mineral Fertilization on Plant Diversity

The diversity index values show a significant effect of fertilization on grassland biodiversity (*p* < 0.001 for all indices). Species richness (S) decreased gradually from 31.75 species in the control variant (V1) to 17.25 species in the intensive mineral fertilization variant (V4). This reduction of more than 45% highlights the eutrophication effect of mineral fertilization on mountain plant communities, leading to the dominance of competitive grasses, particularly *Agrostis capillaris* and *Trisetum flavescens*, at the expense of conservative perennial species such as *Festuca rubra* and *Carex pallescens*. The decline in species richness is associated with the gradual replacement of oligotrophic and conservation-relevant species by mesotrophic and nitrophilous species better adapted to soils with increased nitrogen and phosphorus availability. The Shannon index (H′) followed the same trend, decreasing from 2.62 (Table 6) in the control to 1.62 in V4, confirming the structural simplification of the plant communities as fertilization intensity increased. In contrast, reduced organic fertilization (V2—Low-input) maintained high diversity values (H′ = 2.75), even higher than the control, due to the stimulation of legume species (*Trifolium pratense*, *T. repens*) and mesotrophic species (*Alchemilla vulgaris*), without negative effects on conservation-relevant species. The Evenness index (E) showed high values (>0.8) in the control, organic, and organo-mineral variants, indicating a balanced distribution of species abundances. In contrast, the intensive mineral fertilization variant (V4) exhibited low evenness values (E = 0.57), caused by the pronounced dominance of nitrophilous species (*Agrostis capillaris*, *Trisetum flavescens*). The Simpson index (D) followed the same pattern, with high values (0.89–0.91) in the reduced-input treatments and minimum values (0.63) under intensive fertilization, confirming the decline in α-diversity and the increasing dominance of competitive species. Overall, the results show that moderate organic or organo-mineral fertilization helps maintain floristic balance and preserve the biodiversity of HNV grasslands, whereas intensive mineral fertilization significantly reduces species richness and evenness, promoting structurally simplified eutrophic grassland types.

**Table 6:** The influence of organo-mineral fertilizer on plant diversity.

Variant	Species no. (S)	Shannon (H′)	Evenness (E)	Simpson (D)
V1 (martor)	31.75 ± 0.50 a	2.62 ± 0.08 ab	0.76 ± 0.02 a	0.89 ± 0.02 a
V2 (Low-input)	27.00 ± 0.82 b	2.75 ± 0.05 a	0.84 ± 0.01 a	0.91 ± 0.01 a
V3 (Medium-input)	22.50 ± 1.29 c	2.48 ± 0.06 b	0.80 ± 0.03 a	0.88 ± 0.01 a
V4 (High-input)	17.25 ± 0.50 d	1.62 ± 0.14 c	0.57 ± 0.05 b	0.63 ± 0.05 b
V5 (Organic-mineral)	18.25 ± 0.50 cd	2.38 ± 0.03 b	0.82 ± 0.01 a	0.87 ± 0.01 a
**F test**	239.72	128.01	73.68	85.37
***p*** **Val** **ue**	*p* < 0.001	*p* < 0.001	*p* < 0.001	*p* < 0.001

Notes: Different letters indicate significant differences at *p* < 0.05 (LSD test). The values of the diversity indices (*S*, *H′*, *E*, *D*) show a significant decrease in biodiversity with increased fertilization. Variants with moderate organic fertilization (V2–V3) maintained a balanced floristic structure, while intense mineral fertilization (V4) led to a reduction in species richness and evenness. The results confirm the favorable role of organic and organo-mineral fertilization in maintaining biodiversity in HNV mountain grasslands.

The results highlight the major impact of fertilization type and intensity on the structure and diversity of mountain grasslands, confirming an ecological transition from oligotrophic communities toward mesotrophic and eutrophic grassland types. The MRPP, PCoA, ISA analyses and the diversity indices consistently describe the same trophic gradient driven by progressive fertilization. The following Section 3 addresses the ecological mechanisms underlying these changes and compares the present findings with observations reported for other European mountain ecosystems. These results form the foundation for the ecological interpretation presented in the Section 3.

## 3. Discussion

The results obtained in this study demonstrate that organo-mineral fertilization can represent an effective management strategy for balancing productivity and biodiversity in HNV mountain grasslands of the Apuseni Mountains when applied at appropriate intensity levels. Analyses of floristic composition, diversity indices, and community structure revealed clear contrasts between high mineral input variants, which promoted biomass production at the expense of species diversity, and combined organo-mineral treatments. In particular, the medium-input treatment (V3) maintained moderate productivity while preserving relatively high species richness and Shannon diversity, as the addition of organic matter mitigated the dominance of competitive grasses and reduced the negative impacts of intensive fertilization on grassland vegetation. By comparison, low-input fertilization (V2) primarily supported biodiversity conservation but provided limited productivity gains, underscoring the importance of intermediate fertilization rates in achieving a measurable trade-off between ecosystem services.

### 3.1. The Effects of Fertilization on Floristic Composition and Diversity

Cluster analysis (Sørensen–Bray–Curtis index, UPGMA method) revealed five distinct plant groups, corresponding to a clear trophic gradient determined by fertilization intensity. The succession of these types illustrates the transformation of mountain grasslands under the influence of applied management, from stable oligotrophic stages to eutrophic communities characterized by the dominance of nitrophilic species and reduced floristic diversity, which is useful information for the conservation of biodiversity and the sustainability of HNV mountain ecosystems.

The dominance of *Festuca rubra* in unfertilized treatments reflects the ecological stability of oligotrophic mountain grasslands maintained under long-term extensive management [65]. Such communities are widely described in the literature as biodiversity-rich systems shaped by low nutrient availability and the prevalence of stress-tolerant perennial species [98,99]. Their persistence under zero-input conditions highlights the strong link between nutrient limitation and the maintenance of high floristic diversity, as additional fertilization is known to disrupt competitive balances and favor nutrient-demanding species [100,101]. In this context, *Festuca rubra*–dominated grasslands can be interpreted as conservation-oriented ecological states, characterized by stable species composition and high floristic diversity, indicative of favorable conditions for biodiversity conservation and habitat integrity. Rather than reflecting merely low-productivity systems, these grasslands represent semi-natural communities of high natural value, shaped by long-term low-intensity management and limited nutrient inputs [102,103,104,105].

The coexistence of *Agrostis capillaris* and *Festuca rubra* under moderate organic fertilization reflects a transitional ecological state between oligotrophic and mesotrophic grassland conditions. The increased cover of *Agrostis capillaris*, while *Festuca rubra* remains codominant, suggests that moderate nutrient inputs can alter competitive relationships without disrupting the overall structural integrity of the community. Such grassland types are characteristic of the Apuseni Natural Park and are widely distributed within the region [74]. From a functional perspective, moderate organic fertilization has been shown to enhance functional diversity and improve forage availability while maintaining ecological stability [106], thereby preserving the defining features of habitat 6520—Mountain Meadows [107]. These findings support the broader view that low-input organic fertilization can represent an effective management strategy for enhancing productivity in HNV grasslands without triggering substantial biodiversity loss [11,39,108].

Grassland communities developing under moderate organo-mineral fertilization reflect a balanced ecological response in which productivity gains are achieved without disrupting floristic structure. The coexistence of *Agrostis capillaris*, *Trisetum flavescens*, and *Alchemilla vulgaris* indicates mesotrophic conditions that support both forage production and species diversity. Similar vegetation types have been reported in actively managed mountain hay meadows under moderate fertilization regimes, where ecological stability is maintained despite increased nutrient availability [109,110]. In this context, *Alchemilla vulgaris* is widely recognized as an indicator of mesotrophic and ecologically stable habitats [111,112], suggesting that nutrient inputs and interspecific competition remain within an optimal range. Such mesophilic mountain grasslands represent systems in which trophic inputs are sufficiently high to enhance productivity but not excessive enough to trigger competitive exclusion [19,113]. The observed vegetation responses under moderate organo-mineral fertilization are in line with general trends reported in the literature, which associate moderate nutrient inputs with improved forage quality and dry matter production in mountain grasslands [44,73], supporting the role of this management regime as an effective compromise between agricultural productivity and biodiversity conservation in High Nature Value systems.

In contrast, grasslands receiving exclusively mineral fertilization exhibit a pronounced shift in community structure toward dominance by *Agrostis capillaris*, accompanied by floristic simplification and increased abundance of nitrophilous species. Such patterns have been consistently documented in mineral-fertilized mountain grasslands and are characteristic of ecosystems exposed to high nitrogen availability [11,39,65]. Under these conditions, fast-growing competitive grasses tend to outcompete dicotyledons and species adapted to oligotrophic environments, leading to homogenization of plant communities [114]. Although mineral fertilization may enhance short-term productivity, these changes illustrate its adverse implications for long-term ecological sustainability and the conservation value of HNV grasslands.

At the highest fertilization intensities, grassland communities become increasingly dominated by highly productive mesophilic species, with *Centaurea pseudophrygia* emerging as a codominant element indicative of eutrophic conditions [115,116]. Such communities are characterized by reduced species richness and a more uniform floristic diversity, in line with observations from other intensively fertilized European mountain grasslands [37,117]. The sequence of vegetation responses identified across fertilization treatments thus highlights a clear trophic gradient: moderate organo-mineral inputs maintain ecological balance and floristic diversity, whereas exclusive mineral fertilization and high input levels lead to biodiversity loss and a decline in the ecological value of mountain grasslands.

The contrasting vegetation patterns observed under different fertilization regimes highlight the role of nutrient form and intensity in shaping mountain grassland stability. Mineral-only fertilization promotes floristic simplification by favoring competitive grass species and reducing the presence of oligotrophic taxa typical of nutrient-poor mountain grasslands [72]. Such responses are widely documented and reflect the tendency of high nitrogen inputs to reduce species richness and generate less resilient plant communities with limited resistance to environmental stress [88,118]. In contrast, organo-mineral fertilization supports a more complex and functionally diverse floristic structure, characterized by the persistence of valuable perennial species such as *Trifolium repens*, *Lotus corniculatus*, *Alchemilla vulgaris*, and *Carex pallescens*, which are commonly associated with moderate-input management systems. This pattern aligns with observations from Transylvanian grasslands [38], where mixed fertilization has been shown to enhance ecological stability by maintaining balanced proportions of leguminous and mesotrophic species [69,119]. The higher values of Shannon and Simpson diversity indices under organo-mineral treatments further suggest that reduced reliance on mineral inputs contributes to greater α-diversity and ecosystem stability, supporting the view that combining organic amendments with lower chemical inputs promotes more even and resilient plant communities [82,120].

### 3.2. Ecological Mechanisms and Responses to Organo-Mineral Fertilization

The application of organo-mineral fertilization is widely recognized to influence soil biological functioning and nutrient dynamics through mechanisms reported in previous studies. Although soil microbial parameters were not directly measured in the present study, the observed vegetation responses under combined treatments are consistent with literature indicating that organic inputs can indirectly support nutrient availability and soil functioning by improving soil structure and substrate supply. Therefore, the potential effects on soil biological processes discussed here are interpreted in the context of existing studies rather than as direct experimental evidence from this research [66,84]. The increase in stable organic matter and the gradual release of nutrients contribute to a more balanced nutrient supply, preventing excessive nutrient accumulation and limiting soil structural degradation [106,121]. These processes reduce the risk of competitive exclusion driven by abrupt increases in nutrient availability and help explain the higher species diversity observed under combined fertilization regimes [37,70].

In addition to belowground effects, moderate nutrient availability influences aboveground competitive interactions by limiting excessive biomass production and canopy closure. This reduces light-mediated competition, allowing the persistence of low-growing and stress-tolerant species that are typically excluded under high nutrient conditions [71]. Organic matter inputs further enhance soil water-holding capacity and nutrient retention, buffering plant communities against climatic stress and reducing interannual variability in productivity [122,123].

In contrast, exclusively mineral fertilization, although effective in increasing short-term productivity, often leads to rapid nutrient availability, intensified competition for light, and shifts toward dominance by fast-growing species [37]. Such changes promote trophic imbalance and reduce ecosystem resilience, ultimately resulting in simplified floristic structures and diminished ecological stability [80,123]. Overall, these findings support the concept of a functional compromise, whereby organo-mineral fertilization systems maintain adequate productivity while preserving ecosystem services and floristic diversity in mountain grasslands.

### 3.3. Comparison with Other Studies from the European Mountain Region

The effects of organo-mineral fertilization on the floristic structure observed in the Apuseni Mountains grasslands are consistent with trends reported in the Alpine and Carpathian regions [45,98]. In these areas, the combination of farmyard manure with moderate mineral inputs has maintained high species diversity and a significant proportion of legume species, ensuring a stable pastoral value. At the same time, long-term studies from Transylvania [38,96,124] show that combined fertilization has generated a steady increase in productivity without the loss of species sensitive to eutrophication, confirming the ecological buffering effect provided by organic matter [125]. Our results complement these observations, demonstrating that organo-mineral inputs can be adapted to avoid critical thresholds of intensification that lead to the collapse of diversity.

### 3.4. Implications for the Management of High Nature Value (HNV) Grasslands

From a practical perspective, the results show that moderate organo-mineral fertilization can be effectively integrated into traditional agro-pastoral systems in the Apuseni Mountains, in line with adaptive management principles. The combined use of manure and moderate mineral inputs (below 100 kg N ha^−1^ year^−1^) complies with the Nitrates Directive and Natura 2000 requirements, supporting the maintenance of habitats of Community interest such as *Festuco-Brometalia* and *Nardus stricta* grasslands. Organo-mineral systems therefore contribute to sustainable mountain agriculture by balancing production, biodiversity conservation, and resilience to climate change [67,71,126,127].

Moderate organo-mineral fertilization (≤10 t ha^−1^ manure combined with N_50_P_25_K_25_) maximizes pastoral value without compromising floristic diversity, ensuring both productivity and community stability [37,38]. In contrast, mineral fertilization exceeding 100 kg N ha^−1^ promotes eutrophication, favors nitrophilic species, and reduces species of conservation interest, highlighting the need to limit high input levels. Applying organo-mineral fertilizers mainly in spring improves nitrogen use efficiency by reducing leaching and volatilization, while the use of locally sourced organic fertilizers supports natural nutrient cycles and soil structure. Integrating these practices into Natura 2000 management plans facilitates compliance with EU policies and contributes to the objectives of the EU Biodiversity Strategy 2030 [128,129,130].

Beyond the descriptive patterns observed, the responses of grassland vegetation to fertilization intensity can be explained by well-established ecological mechanisms related to nutrient availability and competitive interactions among plant species. Increased mineral nutrient inputs favor fast-growing, competitive grasses, leading to asymmetric competition for light and a progressive exclusion of less competitive forbs, which explains the reduced species richness under high-input variants. In contrast, the inclusion of organic matter under organo-mineral fertilization likely moderated nutrient release and enhanced spatial and temporal heterogeneity, thereby reducing competitive dominance and allowing the persistence of a more diverse plant community. These mechanisms are consistent with the observed shifts in floristic diversity and community structure along the fertilization gradient and with previous findings from semi-natural mountain grasslands managed under low to moderate input regimes.

While previous studies have documented the effects of organic or mineral fertilization on grassland diversity, most have focused on short- to medium-term responses or on single input types. The present study extends this body of literature by providing evidence from a 15-year long-term experiment that explicitly integrates organic, mineral, and combined organo-mineral fertilization regimes within the same experimental framework. By identifying fertilization thresholds that balance productivity and biodiversity in High Nature Value mountain grasslands, our results offer a more nuanced understanding of how fertilization intensity and nutrient form jointly shape floristic diversity over extended time scales.

This study has some limitations that should be acknowledged. The experiment was conducted at a single site, which may restrict the direct extrapolation of the results to other mountain grassland systems. Although climatic variability can influence grassland dynamics, the present study focuses exclusively on the long-term effects of fertilization gradients under comparable climatic conditions, allowing a clear isolation of fertilization-driven effects on floristic diversity and biodiversity. Future multi-site studies could further explore interactions between fertilization and climatic variability.

## 4. Materials and Methods

This study aims to complement previous research on the effect of fertilization on mountain grasslands, with the objective of analyzing the interactions between organic and mineral fertilization on floristic composition and biodiversity. Unlike the previous study, which focused exclusively on the response to mineral inputs, this paper shows the cumulative effects of organo-mineral fertilization and identifies the indicator species of each trophic regime, providing a more complex perspective on the ecological transition of HNV mountain grasslands.

### 4.1. Study Area

The experiment was carried out in Gârda de Sus (Alba County), within the Apuseni Mountains of the Western Carpathians, Romania (46°29′26.4″ N, 22°48′53.7″ E), on a semi-natural permanent grassland situated at an average altitude of 1130 m and characterized by an approximate slope of 5%. The soil had the typology of red, weakly skeletal preluvosol, with predominantly south-east exposure, characterized by moderate fertility and low content of humus and nutrients, which determined its limited natural productivity [131,132]. The average annual temperature is 5.1 °C (Table 7), and the average annual precipitation is 1042 mm. In 2015, the year analyzed in this study, the average temperature was 7.7 °C, and precipitation was 706.4 mm (Table 8), indicating a warmer and drier season than the multi-year average. The grassland is mowed annually in early July, without grazing. The floristic data were collected after 15 years of differentiated fertilization (2001–2015), reflecting the cumulative effect of organic-mineral fertilization applied annually since 2001, the year the experiment began. Before the experiment was set up in 2001, the pasture was used in a mixed system: it was mowed once a year and grazed extensively by cattle in the fall, with a stocking rate of approximately 0.2–0.4 LU/ha [19,133,134]. In terms of management prior to the experiment, the pasture area was not fertilized, with nutrients coming exclusively from the manure of extensively grazed animals. Starting in 2001, the pasture was maintained exclusively by mowing, followed by the removal of the resulting biomass [19,134].

The research area is located within the Natura 2000, included in the ROSCI0002 Apuseni site, which highlights the importance of conserving rare habitats and species in this area. In particular, the analyzed area belongs to the 6520 Mountain Grassland habitat within the Apuseni Natural Park, a priority habitat for the Natura 2000 network. In this context, our research contributes to a better understanding of how different fertilization regimes can influence the dynamics of a semi-natural mountain ecosystem with high conservation value [11,19,39]. Habitat 6520 in the Apuseni Natural Park is distinguished by its remarkable floristic diversity and exceptional ecological value, the result of the long-term maintenance of traditional grassland use practices. This continuity has allowed the conservation of exceptional flora, including rare species such as *Allium victoriale*, *Anemone narcissifolia*, *Centaurea kotschyana*, *Centaurea mollis*, *Delphinium elatum*, *Dianthus barbatus* ssp. *compactus*, *Hypericum richerii* ssp. *grisebachii*, *Lilium jankae*, *Trollius europaeus* [22,105,106,107,108]. In this complex floristic setting, which is sensitive to changes in nutrients, the results of the present study become relevant for establishing adaptive management practices that ensure both productivity and long-term conservation of HNV grasslands in the Apuseni Mountains.

### 4.2. Experimental Design

Data used to analyze vegetation trends were obtained from a long-term experiment initiated in 2001. A completely randomized block design was used, with 5 treatment variants and 4 replications, on a total of 20 adjacent plots, each with an area of 20 m^2^ (2 × 10 m). Within each block, treatments were distributed among the experimental plots following a predefined randomized layout. Spatial independence among blocks was ensured by 1 m buffer zones separating the four replications. The variants were as follows (kg/ha): T1—control variant (*Zero-input*); T2—10 t/ha^−1^ manure (*Low-input organic*); T3—10 t/ha^−1^ manure + N_50_P_25_K_25_ (*Medium-input organo-mineral*); T4—N_100_P_50_K_50_ (*Medium-input mineral*); T5—10 t/ha^−1^ manure + N_100_P_50_K_50_ (*High-input organo-mineral*). Manure from local farms (cattle and horses) had, on average, the following composition (kg t^−1^ dry matter): 0.40 N, 0.39 P, and 0.45 K. The composition of the organic manure was verified annually through laboratory analyses of representative samples from the local farms. Complex mineral fertilizers (NPK 20:10:10) were applied in the spring, before the start of vegetation. Treatments were applied annually in each experimental variant. Mowing was performed at the optimal stage, corresponding to the phenological flowering phase of grasses, and the harvested biomass was removed from the experimental area. The entire experimental field was fenced to prevent access by wild or domestic animals. Prior to the establishment of the experiment, a detailed floristic survey of the meadow vegetation was conducted in 2001. All plant species present within the experimental area were identified, and their cover was assessed, thereby defining the initial floristic structure (Moment 0) before plot delimitation and the subsequent application of experimental treatments later in 2001. The experiments were established on a brown eu-mezo-bazic rendzinic soil, characterized at the initial stage by a pH ranging from 5.21 in the At horizon to 6.70 in the Bv2 horizon, and by a humus content decreasing with depth, from 15.42% in the At horizon to 2.61% in the Bv2 horizon [134].

### 4.3. Floristic Diversity

**Figure 3:**
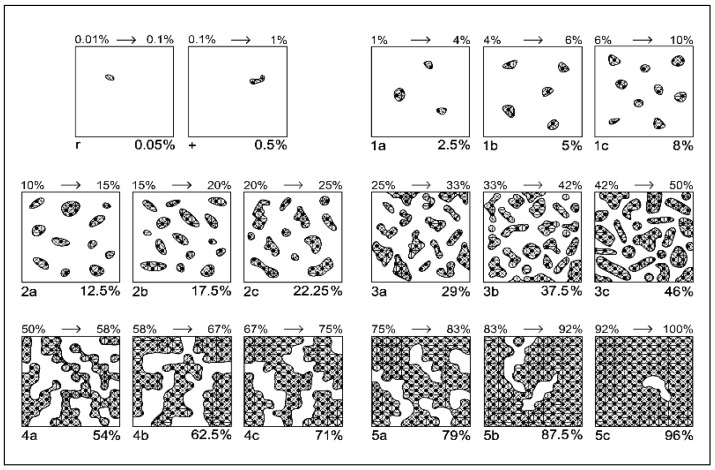
Modified Braun-Blanquét scale for grasslands based on species coverage (after [92]). Legend: 1 to 5 indicate the class of coverage; a, b, c indicate the sub-note of each class.

Floristic diversity was evaluated using a modified Braun–Blanquet method adapted for mountain grasslands by [92] (Figure 3, Table 9). A comparable methodological approach has been applied in recent studies from the Eastern Alps (e.g., case studies conducted in three mountain regions), where the Braun–Blanquet scale was subdivided into finer cover classes to enhance assessment resolution and improve the accuracy of species cover estimates in alpine grasslands. This refinement is justified by the need for more precise discrimination of cover variations, particularly in plant communities with complex structure and high species diversity, such as HNV mountain grasslands. The cover estimation was carried out in early July, when most grasses were in the flowering phase. This harvesting period ensured relevance for comparisons regarding vegetation structure. Floristic studies were carried out in each experimental variant on the entire 20 m^2^ area, with 4 repetitions each, and this paper presents floristic data over a period of 1 years (2015), which reflects the cumulative effect of organo-mineral fertilization after 15 years of application. Plant species were identified using specialized identification keys and appropriate field instruments. Scientific nomenclature followed internationally recognized botanical resources and databases, including Plants of the World Online (POWO) and the Euro + Med PlantBase [19]. To ensure data accuracy, all experimental variants were fenced to prevent access by animals that could influence the study outcomes. Floristic surveys were conducted at the optimal period for vegetation assessment in the study area, namely July, when grasses are in full bloom, a timing that ensures reliable species identification and composition estimates, as demonstrated by previous studies on semi-natural grasslands [135,136].

**Table 8:** Monthly mean precipitation recorded at the Ghețari meteorological station (2015 year).

Year	Months												Total
	I	II	III	IV	V	VI	VII	VIII	IX	X	XI	XII	
2015	32.6	14.2	23.6	48.6	69.4	78.2	33.8	95	124	38.8	98.4	49.8	706.4
**Average values calculated for the period 2001–2017**													
2001–2017	67.2	55.9	81.8	77.2	102.4	100.3	137.3	98.4	92.6	86.6	86.5	55.8	1042.1

**Table 9:** Modified Braun-Blanquét scale for assessing the abundance–dominance of plant species, based on classes and sub-classes (after [92]).

Class	Coverage Interval (%)	Class Central Value (%)	Sub-Note	Sub-Interval (%)	Central-Adjusted Value of Sub-Interval (%)
5	75–100	87.5	5 c	92–100	96
			5 b	83–92	87.5
			5 a	75–83	79
4	50–75	62.5	4 c	67–75	71
			4 b	58–67	62.5
			4 a	50–58	54
3	25–50	37.5	3 c	42–50	46
			3 b	33–42	37.5
			3 a	25–33	29
2	10–25	17.5	2 c	20–25	22.25
			2 b	15–20	17.5
			2 a	10–15	12.5
1	1–10	5	1 c	6–10	8
			1 b	4–6	5
			1 a	1–4	2.5
+	0.1–1	0.5	-	-	0.5
r	0.01–0.1	0.05	-	-	0.05

Note: a, b, c indicate the sub-note of each class.

### 4.4. Data Analysis

Multivariate analyses were performed using PC-ORD v.7 [137,138], a statistical package widely used in community ecology for classification, ordination, and testing differences among groups. The software has proven highly effective for examining complex floristic structures [139]. To classify floristic types, cluster analysis was applied, based on the Sørensen (Bray–Curtis) index and the UPGMA agglomeration method, both regarded as standard approaches in plant community analysis [140,141]. Bray–Curtis (Sørensen) dissimilarity was calculated based on relative species cover data, obtained by transforming the modified Braun–Blanquet abundance–dominance classes into percentage cover values. The dendrogram cut level was set to retain approximately 80% of the information, which allowed the identification of groups with clear ecological and phytosociological relevance. To highlight the trophic gradient and differentiate grassland types according to treatment, Principal Coordinates Analysis (PCoA) was used, based on Bray–Curtis distances—an established method in plant community ecology for comparing floristic and microbial compositions [121,142]. The choice of PCoA is justified by its ability to provide a unique and reproducible solution, allowing a straightforward interpretation of differences among groups, unlike methods such as NMDS, which may yield variable solutions. The combined use of PCoA and Bray–Curtis distance is well supported in the recent literature for assessing fertilization-induced changes in plant, fungal, and bacterial communities [143]. All multivariate analyses (cluster analysis, PCoA, MRPP, and Indicator Species Analysis) were performed using PC-ORD version 7.

For each experimental variant, vectors corresponding to the fertilization levels were defined as follows: *ZeroInput* (T1—control, no fertilization), *LowOrg* (T2—10 t/ha^−1^ manure), *MedOrgMin* (T3—10 t/ha^−1^ manure + N_50_P_25_K_25_), *MedMin* (T4—N_100_P_50_K_50_), and *HighOrgMin* (T5—10 t/ha^−1^ manure + N_100_P_50_K_50_). The classification of treatments according to input intensity (*“Zero-”*, *“Low-”*, *“Medium-”*, *“High-input*”) follows the usual practice in experimental ecology studies, where different levels of fertilization are compared to assess the effects on biodiversity and grassland productivity [121]. This structure complies with the thresholds set by the Nitrates Directive (170 kg N ha^−1^ year^−1^) and reflects the ecological response observed in the field. The vectors were normalized and tested using Monte Carlo permutations (n = 999) to verify statistical significance. The use of experimental vectors in PCoA analysis is in line with methodological recommendations for studies based on Bray–Curtis distance and joint plot ordination [144]. Ordination axes were interpreted based on the percentage of variance explained, whereas Pearson correlation coefficients (r) were used only for species–axis and treatment–axis relationships. The first two axes were retained for interpretation, as they explained over 97% of the total variation in floristic diversity. To verify the differences between the groups obtained by cluster analysis and PCoA, the MRPP (*Multi-Response Permutation Procedure*) was applied, a non-parametric method widely used in plant community ecology. This method compares the similarity between groups and provides three main indicators: the A coefficient, which expresses the internal homogeneity of the groups (positive values indicate greater consistency than expected); the T statistic, which reflects the degree of separation between groups (more negative values indicate clearer differentiation); and the *p* value, which assesses the statistical significance of the differences. The significance threshold was set at α = 0.05, according to standards in ecological community analysis. The application of MRPP is frequently reported in the recent literature, being used to validate the separation of plant or animal communities according to experimental treatments and environmental factors [145]. In this study, grassland trophic categories were defined using ecological indicator values for nitrogen following [92]. Oligotrophic grasslands refer to plant communities dominated by species adapted to nutrient-poor soils, characterized by low nitrogen availability and low indicator values. Mesotrophic grasslands represent intermediate conditions, where species with moderate nutrient requirements prevail, reflecting balanced soil fertility. Eutrophic grasslands correspond to nutrient-rich conditions, dominated by nitrophilous species associated with high nitrogen availability. These categories were used as functional ecological descriptors and were assigned based on the prevailing nitrogen indicator values of species and their correspondence with the applied fertilization gradient [92].

To identify the species characteristic of each fertilization regime, Indicator Species Analysis (ISA) was applied, according to the methodology of [146]. This procedure combines the fidelity and constancy of species occurrence within treatments, generating an indicator importance value (IV, %) for each species. The statistical significance of the IV values was assessed by permutation tests (n = 999), a method recognized for its accuracy in highlighting flora-environment relationships and structural changes caused by fertilization [146,147].

### 4.5. Diversity Index Analysis

For each experimental treatment, α-diversity indices were computed, including species richness (S), the Shannon–Wiener index (H′), species evenness (E), and the Simpson index (D). These indices are widely applied in grassland ecological studies and are regarded as reliable measures for evaluating community diversity and structural balance [19,148,149]. Their applicability has been further demonstrated by studies that used these metrics to characterize vegetation diversity from remote sensing data [150], as well as by investigations reporting significant differences between plant communities in protected versus unprotected areas [19,151], thereby confirming their relevance for ecosystem analysis. Calculation formulas are well established in community ecology [91] and were applied using species relative abundance data. Differences among treatments were tested using one-way ANOVA, and mean separation was performed with the LSD test at a significance level of *p* < 0.05 [152].

## 5. Conclusions

This long-term study demonstrates that fertilization intensity and regime are key drivers of floristic diversity and biodiversity in HNV mountain grasslands. Moderate organo-mineral fertilization maintains a favorable balance between productivity and species diversity, whereas exclusive mineral fertilization and high input levels lead to floristic simplification and reduced conservation value.

From a management perspective, the results support the use of low to moderate organo-mineral inputs (≤10 t ha^−1^ manure combined with N_50_P_25_K_25_) as a practical strategy for sustaining HNV grasslands. Such regimes enhance pastoral value while preserving structurally diverse and species-rich plant communities, avoiding the ecological degradation associated with intensive fertilization.

At the policy level, these findings are directly relevant to the implementation of the Nitrates Directive and Natura 2000 management objectives, providing empirical support for fertilization thresholds that reconcile agricultural use with biodiversity conservation.

Future research should expand this approach through multi-site experiments and long-term monitoring, with particular attention to soil biological processes and plant–soil interactions, in order to better understand the mechanisms underlying ecosystem resilience under different fertilization regimes.

## Figures and Tables

**Table 1:** Importance of axis.

Axis	Explained Variance (%)	Cumulative Variance (%)
1 (r) (X)	73.1	73.1
2 (r) (Y)	23.9	97.0

Note: r—represents Pearson correlation coefficients between species or treatment vectors and ordination axes.

**Table 2:** Correlation of treatment variants with ordination axis.

Experimental Factors	Axis 1 (r)	Significance	Axis 2 (r)	Significance
ZeroInput (T1)	−0.922	***	0.258	ns
LowOrg (T2)	−0.092	ns	−0.487	*
MedOrgMin (T3)	0.168	ns	−0.385	*
MedMin (T4)	0.419	*	0.846	***
HighOrgMin (T5)	0.427	*	−0.233	ns

Note: *r*—Pearson correlation coefficient between treatment vectors and ordination axes. Significance. *p* ˂ 0.001—***; *p* ˂ 0.5—*; ns—not significant. *ZeroInput*—T1- control variant—*Zero-input*; *LowOrg*—T2—10 t/ha^−1^ manure—*Low-input organic*, *MedOrgMin*—T3—10 t/ha^−1^ manure+ N_50_P_25_K_25_—*Medium-input organic mineral*, *MedMin*—T4—N_100_P_50_K_50_—*Medium-input mineral*, *HighOrgMin*—T5—10 t/ha^−1^ manure + N_100_P_50_K_50_—*High-input*.

**Table 3:** Comparison of the floristic composition changes due to organo-mineral inputs (MRPP).

Treatments	T	A	*p* Value
T1 vs. T2	−4.442	0.667	0.0056 **
T1 vs. T3	−4.463	0.703	0.0056 **
T1 vs. T4	−4.467	0.741	0.0056 **
T1 vs. T5	−4.464	0.739	0.0056 **
T2 vs. T3	−4.407	0.489	0.0056 **
T2 vs. T4	−4.464	0.697	0.0056 **
T2 vs. T5	−4.422	0.527	0.0055 **
T3 vs. T4	−4.450	0.650	0.0056 **
T3 vs. T5	−4.348	0.428	0.0057 **
T4 vs. T5	−4.431	0.593	0.0056 **

Note. T—test statistic, A—agreement statistic. Significance. *p* < 0.01—**; ns—not significant. The results of the *Multi-Response Permutation Procedure* (MRPP) test highlight significant differences among all analyzed treatments (*p* < 0.01). The negative values of the T statistic and the positive A coefficients indicate clear separation between groups and high internal homogeneity. The most pronounced differentiation between plant communities was observed between the control variant (T1) and those fertilized with mineral (T4) or organo-mineral inputs (T3), confirming the significant influence of nutrient supply on floristic composition. The agreement statistic (A) represents the effect size, with values <0.1 indicating weak separation, 0.1–0.3 moderate separation, and values >0.3 indicating strong separation among groups. T1 control variant—*Zero-input*; T2—10 t/ha^−1^ manure—*Low-input organic*, T3—10 t/ha^−1^ manure+ N_50_P_25_K_25_—*Medium-input organo-mineral*, T4—N_100_P_50_K_50_—*Medium-input mineral*, T5—10 t/ha^−1^ manure + N_100_P_50_K_50_—*High-input.*

**Table 4:** Correlation of species with the ordination axis.

Species	Axis 1				Axis 2			
	r	r-sq	tau	Signif.	r	r-sq	tau	Signif.
*Agrostis capillaris* L.	0.787	0.620	0.793	***	0.584	0.342	0.318	**
*Anthoxanthum odoratum* L.	−0.592	0.350	−0.628	**	−0.382	0.146	−0.405	*
*Cynosurus cristatus* L.	−0.691	0.477	−0.711	***	−0.501	0.251	−0.474	**
*Festuca rubra* L.	−0.952	0.907	−0.800	***	0.043	0.002	−0.035	ns
*Trisetum flavescens* (L.) P. Beauv.	0.300	0.090	0.108	*	−0.694	0.481	−0.672	***
*Carex pallescens* L.	−0.691	0.477	−0.711	***	−0.501	0.251	−0.474	**
*Luzula multiflora* (Ehrh.) Lej.	−0.691	0.477	−0.711	***	−0.501	0.251	−0.474	**
*Lotus corniculatus* L.	−0.828	0.685	−0.711	***	−0.187	0.035	−0.207	ns
*Trifolium pratense* L.	−0.213	0.045	−0.288	ns	−0.503	0.253	−0.428	**
*Trifolium repens* L.	0.136	0.019	−0.120	ns	−0.783	0.614	−0.799	***
*Vicia cracca* L.	0.383	0.147	0.177	*	−0.666	0.444	−0.598	**
*Alchemilla vulgaris* L.	−0.507	0.257	−0.408	**	−0.192	0.037	−0.124	ns
*Campanula abietina* Griseb.	−0.922	0.849	−0.580	***	0.258	0.066	0.290	ns
*Carlina acaulis* L.	−0.840	0.705	−0.617	***	−0.003	0.000	−0.032	ns
*Centaurea pseudophrygia* C.A. Mey.	0.288	0.083	0.148	ns	−0.742	0.550	−0.549	***
*Cerastium holosteoides* Fr.	−0.922	0.849	−0.580	***	0.258	0.066	0.290	ns
*Colchicum autumnale* L.	0.117	0.014	−0.058	ns	−0.887	0.786	−0.759	***
*Euphrasia carnica* Wettst.	−0.531	0.282	−0.250	**	0.111	0.012	0.222	ns
*Hypericum maculatum* Crantz	0.291	0.085	0.361	*	0.826	0.682	0.754	***
*Leucanthemum vulgare* Lam.	−0.693	0.480	−0.708	***	0.050	0.003	−0.150	ns
*Pimpinella major* (L.) Huds.	0.795	0.632	0.573	***	−0.265	0.070	−0.140	ns
*Plantago lanceolata* L.	−0.925	0.856	−0.824	***	0.082	0.007	−0.267	ns
*Plantago media* L.	−0.889	0.791	−0.576	***	0.233	0.054	0.257	ns
*Polygala vulgaris* L.	−0.922	0.849	−0.580	***	0.258	0.066	0.290	ns
*Potentilla erecta* (L.) Raeusch.	−0.910	0.828	−0.680	***	0.262	0.069	0.095	ns
*Ranunculus acris* L.	−0.086	0.007	−0.079	ns	−0.156	0.024	−0.063	ns
*Ranunculus bulbosus* L.	−0.056	0.003	−0.092	ns	−0.052	0.003	−0.025	ns
*Rhinanthus minor* L.	−0.922	0.849	−0.580	***	0.258	0.066	0.290	ns
*Rumex acetosa* L.	0.653	0.427	0.613	**	0.056	0.003	−0.012	ns
*Stellaria graminea* L.	0.245	0.060	0.053	ns	−0.576	0.332	−0.525	**
*Taraxacum officinale* Weber	0.617	0.381	0.457	**	−0.572	0.327	−0.226	**
*Thymus pulegioides* L.	−0.922	0.849	−0.580	***	0.258	0.066	0.290	ns
*Tragopogon pratensis* L.	−0.645	0.416	−0.522	**	−0.172	0.030	−0.190	ns
*Veronica chamaedrys* L.	0.728	0.529	0.485	**	−0.391	0.153	−0.138	ns
*Viola declinata* Waldst. & Kit.	−0.828	0.685	−0.711	***	−0.187	0.035	−0.207	ns

Note: *r*—correlation coefficient; *r-sq*—determination coefficient. Significance. *p* ˂ 0.001—***; *p* ˂ 0.01—**; *p* ˂ 0.05—*; ns—not significant. Negative r-values are linked to conservative species (*Festuca rubra*, *Carex pallescens*, *Luzula multiflora*), whereas positive values correspond to species adapted to mineral and organo-mineral fertilization (*Agrostis capillaris*, *Trisetum flavescens*, *Rumex acetosa*). Consequently, Axis 1 reflects the intensity of fertilization, while Axis 2 differentiates the type of nutrient input (organic vs. mineral inputs).

**Table 5:** The Indicator Species Analysis (ISA) highlighted species with a significant affinity for each fertilization regime.

Species	Max Group	Indicator Value (IV)	Mean	S.Dev	*p*-Value
*Agrostis capillaris* L.	4	40.9	27.5	3.14	** (*p* = 0.0014)
*Anthoxanthum odoratum* L.	1	29.1	27.4	3.12	ns (*p* = 0.5537)
*Cynosurus cristatus* L.	1	33.3	25.6	7.29	ns (*p* = 0.4681)
*Festuca rubra* L.	1	45.7	29.8	4.10	** (*p* = 0.0012)
*Trisetum flavescens* (L.) P. Beauv.	3	55.9	32.9	5.57	** (*p* = 0.0012)
*Carex pallescens* L.	1	33.3	25.6	7.29	ns (*p* = 0.4681)
*Luzula multiflora* (Ehrh.) Lej.	1	33.3	25.6	7.29	ns (*p* = 0.4681)
*Lotus corniculatus* L.	1	50.0	24.7	9.84	* (*p* = 0.0476)
*Trifolium pratense* L.	2	41.0	30.7	4.29	* (*p* = 0.0470)
*Trifolium repens* L.	2	43.8	30.8	4.47	* (*p* = 0.0220)
*Vicia cracca* L.	3	34.7	28.3	3.79	ns (*p* = 0.1688)
*Alchemilla vulgaris* L.	3	32.1	25.9	2.45	* (*p* = 0.0420)
*Campanula abietina* Griseb.	1	100.0	22.2	12.47	** (*p* = 0.0012)
*Carlina acaulis* L.	1	66.7	23.3	10.76	* (*p* = 0.0148)
*Centaurea pseudophrygia* C.A. Mey.	5	33.9	27.3	3.01	ns (*p* = 0.0552)
*Cerastium holosteoides* Fr.	1	100.0	22.2	12.47	** (*p* = 0.0012)
*Colchicum autumnale* L.	2	32.5	27.7	4.20	ns (*p* = 0.3235)
*Euphrasia carnica* Wettst.	1	66.7	29.9	12.37	* (*p* = 0.0368)
*Hypericum maculatum* Crantz	4	32.8	25.2	2.17	** (*p* = 0.0054)
*Leucanthemum vulgare* Lam.	1	66.7	29.9	12.53	* (*p* = 0.0370)
*Pimpinella major* (L.) Huds.	5	29.4	24.7	1.84	* (*p* = 0.0342)
*Plantago lanceolata* L.	1	74.5	31.8	10.33	** (*p* = 0.0016)
*Plantago media* L.	1	100.0	22.7	12.55	** (*p* = 0.0012)
*Polygala vulgaris* L.	1	100.0	22.2	12.47	** (*p* = 0.0012)
*Potentilla erecta* (L.) Raeusch.	1	74.5	35.5	9.44	** (*p* = 0.0012)
*Ranunculus acris* L.	1	16.1	26.2	4.91	ns (*p* = 1.0000)
*Ranunculus bulbosus* L.	2	26.7	25.8	3.07	ns (*p* = 0.9264)
*Rhinanthus minor* L.	1	100.0	22.2	12.47	** (*p* = 0.0012)
*Rumex acetosa* L.	5	39.4	29.9	6.09	ns (*p* = 0.0782)
*Stellaria graminea* L.	3	33.3	29.3	4.79	ns (*p* = 0.4803)
*Taraxacum officinale* Weber	5	41.1	28.8	5.07	* (*p* = 0.0234)
*Thymus pulegioides* L.	1	100.0	22.2	12.47	** (*p* = 0.0012)
*Tragopogon pratensis* L.	1	37.5	23.4	10.72	ns (*p* = 0.3069)
*Veronica chamaedrys* L.	5	35.6	27.6	3.05	* (*p* = 0.0174)
*Viola declinata* Waldst. & Kit.	1	50.0	24.7	9.84	* (*p* = 0.0476)

Notes. Significance. *p* ˂ 0.01—**; *p* ˂ 0.5—*; ns—not significant. *Max group*—treatment with maximum indicator value, 1—control variant; 2—10 t/ha^−1^ manure; 3—10 t/ha^−1^ manure+ N_50_P_25_K_25_; 4—N_100_P_50_K_50_; 5—10 t/ha^−1^ manure + N_100_P_50_K_50_*; IV*—indicator value *(%)*; *S.Dev*—standard deviation. *Indicator species analysis (ISA)* revealed significant associations between dominant species and different fertilization regimes. The control variants were characterized by oligotrophic species (*Festuca rubra*, *Campanula abietina*, *Thymus pulegioides*), while moderate fertilization favored mesotrophic species (*Trisetum flavescens*, *Alchemilla vulgaris*). High fertilization, whether mineral or organo-mineral, was associated with nitrophilous and eutrophic species (*Agrostis capillaris*, *Centaurea pseudophrygia*), confirming the trophic gradient determined by the intensification of inputs.

**Table 7:** Monthly mean air temperatures recorded at the Ghețari meteorological station (2015 year).

Year	Months												Average
	I	II	III	IV	V	VI	VII	VIII	IX	X	XI	XII	
2015	−0.8	2	4.7	3.8	10.8	13.5	16.5	16.5	12.7	7.7	3.4	−0.2	7.7
**Average values calculated for the period 2001–** **2017**													
2001–2017	−4.5	−2.7	0.2	5.3	10.5	14.5	15.9	15.4	11.3	6.0	1.4	−3.1	5.8

## Data Availability

The original contributions presented in this study are included in the article. Further inquiries can be directed to the corresponding author.
